# Insights into the gut microbiota characteristics between the organic and traditional feeding chickens based on amplicon and metagenomic sequencing

**DOI:** 10.3389/fmicb.2024.1509461

**Published:** 2025-01-23

**Authors:** Wenzhou Zhang, Xiaoru Jian, Siqi Ding, Jiamin Chang, Shouping Ji, Yulang Chi

**Affiliations:** ^1^School of Pharmacy, QuanZhou Medical College, Quanzhou, China; ^2^College of Oceanology and Food Science, Quanzhou Normal University, Quanzhou, China; ^3^Fujian Province Key Laboratory for the Development of Bioactive Material from Marine Algae, Quanzhou Normal University, Quanzhou, China

**Keywords:** intestinal microorganisms, organic chickens, feed chickens, metagenome technology, microbial diversity

## Abstract

Intestinal microorganisms play a crucial role in chicken health and production performance, especially in the research of traditional and organic feeding methods. The intestinal contents of organic and feed chickens were analyzed by 16S rRNA gene and metagenome technology. The results showed that the microbial diversity of organic chickens was significantly higher than that of the feed chickens, especially the key microorganisms, such as *Enterococcus*, were more abundant in organic chickens. The functional analysis of metagenome revealed the significant difference in the metabolic function of intestinal microorganisms between them. The present study provides new insights into the gut microbiota characteristics of the organic and feed chicken based on amplicon and metagenomic sequencing. Our results are helpful to fully illustrate the effects of different feeding methods on intestinal microorganisms in chickens and can offer a more scientific basis for chicken production management.

## 1 Introduction

Poultry meat is a significant component of human diets worldwide, but the impact of feeding systems on the quality and productivity of chicken products still remains controversial (Dong et al., 2024). In recent years, gut microbiota has attracted much attention due to its important role in intestinal development and metabolic homeostasis. Studies have found that the gut microbes of mammals play an important role in food digestion, vitamin and amino acid synthesis, organ development, and host physiological regulation (Kers et al., 2018). However, the gut microbial composition of chickens is influenced by various factors such as diet, climate, and geographical location (Dai et al., 2023; Feng et al., 2023). The chicken intestinal microbe is an important part of the chicken digestive system, which has an important effect on chicken health and production performance (Borda-Molina et al., 2018). With the introduction of different feeding methods, including traditional feeding and organic feeding, more and more researches have been done on chicken gut microbes (Sun et al., 2018; Bernard et al., 2024; Zhou et al., 2016). In recent years, intestinal microbiome studies in organic and feed chickens have attracted much attention, especially using 16S rRNA gene amplicon and metagenomics techniques (Sun et al., 2018). Through 16S rRNA gene sequencing, the microbial structure and community members could be rapidly identified and classified. It was found that the composition and structure of intestinal microbial communities due to different feeding methods were significantly different, which were affected by some factors such as feeding environment, feed, and growth stage. At the same time, the high probiotic content of organic chickens helps balance gut microbes, while forage chickens are more susceptible to pathogenic bacteria (Chen et al., 2023). Metagenomics studies further revealed their functional and metabolic potential, found differences in metabolic pathways and functional genes, and provided the scientific basis for optimizing feed formulation, improving feeding management, and promoting sustainable development of poultry production.

There is a close relationship between chicken intestinal microbes and chicken quality, which is mainly reflected in chicken health, breeding efficiency, and meat quality (Chen et al., 2023; Young et al., 2007; Stanley et al., 2014). The gut microbiome plays a crucial role in the overall health of chickens. In a good feeding environment, maintaining a normal intestinal microecological balance is very important to promote the digestive function of chickens and improve the feed utilization rate. The gut microbiota plays a crucial role in the distribution of nutrients in chickens in multiple ways. Some beneficial gut microbiota can participate in the breakdown and fermentation of complex carbohydrates, such as converting indigestible substances like cellulose into absorbable nutrients like short chain fatty acids. These short chain fatty acids not only provide energy for the intestinal epithelial cells of chickens, maintaining the integrity of the intestinal barrier function, but also can be absorbed into the bloodstream, affecting the regulation of energy metabolism throughout the body. A good intestinal microecological environment contributes to a healthy growth environment and promotes the rational distribution of nutrients in the chicken, which may lead to more uniform muscle development and higher meat quality (Sun et al., 2018). In contrast, affected by intestinal microecological imbalance, the distribution of nutrients in chickens may be uneven, affecting the consistency and taste of meat quality. Therefore, scientific management of feed and feeding conditions to maintain intestinal microecological balance is crucial to improve chicken breeding efficiency and meat quality. A good intestinal microecological environment can also improve the disease resistance of chickens. The normal gut microbiota inhibits the growth of harmful microorganisms, forming a natural protective barrier against pathogenic microorganisms and reducing the risk of disease in chickens (Kers et al., 2018; Aruwa et al., 2021; Sun et al., 2016). This has a positive impact on reducing the use of drugs, reducing antibiotic residues, and producing healthier chicken. Adopting reasonable feeding and management measures to ensure a good intestinal microecological environment for chickens, not only helps to guarantee the overall health of chickens and improve breeding efficiency but also can produce more high-quality and safe chickens to meet the needs of consumers for food quality and safety (Bernard et al., 2024; Oviedo-Rondón, 2019). Therefore, paying attention to and maintaining the health of chicken intestinal microbes has become an important link that cannot be ignored in modern poultry farming.

There are significant differences between the organic and feed chickens, and the effects on chicken quality can be assessed by analyzing the gut microbial community of chickens (Coletta et al., 2012). Herein, the effects of different feed methods on intestinal flora were analyzed by sampling the intestinal contents of chickens. The difference in intestinal bacterial community between organic and feed chickens was studied by 16S rRNA gene full-length amplification analysis. To determine the effects of different farming practices, the function of chicken gut microbes was studied based on metagenomic sequencing. The results of this study provide a new way to study the diversity of intestinal microflora of organic and feed chickens and its effects on chicken quality, aiming to deeply understand the effects of feeding methods on chicken intestinal microflora, and provide a more scientific basis for chicken production management. Research can explore how to improve the gut microbiota of chickens by optimizing feed formulations and feeding patterns, thereby enhancing the sustainability and economic benefits of organic agriculture. For example, by using organic feed raw materials to reduce chemical dependence, lower environmental pollution risks, and improve animal health.

## 2 Materials and methods

### 2.1 Animals feeding and sample collection

All animal experiments were approved by the Research Ethics Committee of Quanzhou Normal University (Approval No. QZTC-LL-2023-598). The domestic male chickens (*Gallus gallus*) were purchased from a poultry farm. These chickens are about 30 days old. They were randomly divided into two groups, each group containing 20 chickens, which were cereal fed chickens (CE) and forage fed chickens (FO). Each group of chickens was confined to a fixed cage (3.0 × 2.0 × 1.0 m). In terms of diet, CE chickens are fed with rice, and FO chickens are fed with commercial feed (Table 1) (Al-Khalaifah and Al-Nasser, 2021). Each cage is equipped with a water bottle. The water is changed every 2 days and the chickens can drink as much as they like. Other feeding and management conditions are exactly the same. No antibiotic drugs or probiotic products were used throughout the feeding trial (Sun et al., 2018). Antibiotics may kill beneficial commensal bacteria in the gut, affecting the efficiency of digesting and absorbing nutrients in food. This may lead to abnormal changes in the growth performance, body composition and other indicators of the experimental animals, which are not caused by the feeding factors studied (such as feed formula, feeding mode, etc.), but by the side effects of antibiotics.

**Table 1:** The commercial feed composition.

Name	Content
Crude protein	≥20.0
Crude fiber	≤6.0
Crude ash	≤8.0
Calcium	≥0.2–1.0
Total phosphorus	≥0.5
Sodium chloride	0.20–0.80
Egg + cystine	≥0.74
Moisture	≤14.0

At 360 days old, six chickens of similar weight were selected from each group and the intestinal contents were collected. The intestinal contents were collected under sterile conditions and then stored in liquid nitrogen for later gut microbiota analysis.

### 2.2 DNA extraction, PCR amplification, and 16S Illumina sequencing

The intestinal contents of 12 chickens (CE = 6, FO = 6) were collected, and DNA samples were extracted by the CTAB method. The purity and concentration of DNA samples were detected by 1% concentration agarose gel electrophoresis (Ding et al., 2024). The V3 to V4 variable region of bacterial 16S rRNA gene was amplified with the universal primer 314F (5′-CCTAYGGGRBGCASCAG-3′) and 806R (5′-GGACTACNNGGGTATCTAAT-3′). The PCR amplification reaction process is first heated at 95°C for 1 min to denature the first DNA of the template, followed by 30 cycles at 98°C (10 s), 50°C (30 s), 72°C (30 s), and finally held at 72°C for 5 min (Minas et al., 2011). Sequencing libraries were generated using NEB Next^®^ Ultra DNA Library Prep Kit (Illumina, United States) following the manufacturer’s recommendations and index codes were added. The library quality was assessed on the Agilent 5400 (Agilent Technologies Co Ltd., United States). At last, the library was sequenced on an Illumina platform and 250 bp paired-end reads were generated.

### 2.3 Bioinformatic analysis based on 16S rRNA full-length amplicon sequencing

The analysis followed the Qiime2docs “Atacama soil microbiome tutorial” and utilized customized program scripts. Raw data FASTQ files were formatted for QIIME2, demultiplexed, quality filtered, de-noised, merged, and had chimeric sequences removed using the QIIME2 dada2 plugin to create an amplicon sequence variant (ASV) feature table (Guan et al., 2022). ASV sequences were aligned to a pre-trained GREENGENES database for taxonomy classification. Contaminating sequences were filtered, and various statistical methods were applied for abundance analysis. Diversity metrics were calculated, and beta diversity distances were measured using Bray Curtis, unweighted UniFrac, and weighted UniFrac metrics. PLS-DA and redundancy analysis were used to analyze microbiota variation and community associations with environmental factors. Co-occurrence analysis was conducted, and PICRUSt was utilized to predict microbial functional profiles. Default parameters were used unless specified otherwise.

### 2.4 Metagenome sequencing analysis

Based on the results of 16S rRNA, three samples from each group were selected for metagenome sequencing analysis. The raw data of bacteria, fungi, and viruses in the sample of 6 chickens (CE = 6, FO = 6) were obtained by metagenomic sequencing using the Illumina Novaseq high-throughput sequencing platform (Franzosa et al., 2018). In order to ensure the reliability of data, the raw sequencing data needs to be preprocessed using Kneaddata software. The clean reads after quality control and de-host were used to be blasted to database (Uniref90) using Humann2 software (based on Diamond), and the annotation information and relative abundance table from each functional database were obtained according to the corresponding relationship between Uniref90 ID and each database. Based on the species abundance table and functional abundance table, abundance clustering analysis, PCOA and NMDS dimension reduction analysis (species only), and sample clustering analysis can be performed. When grouping information is available, Lefse Biomarker and Dunntest analysis can be performed to excavate differences in species composition and functional composition between samples.

### 2.5 Statistical analysis

In this study, data are expressed as mean ± SD, and independent sample t-tests were conducted using IBM SPSS Statistics 20 to evaluate significant differences between groups. Differences were deemed statistically significant if the *p*-value was less than 0.05 (*p < 0.05, **p < 0.01, ***p < 0.001). Statistical analysis and data plotting were performed with GraphPad Prism 8 (GraphPad, La Jolla, California, United States) or Wekemo Bioincloud.^1^

## 3 Results

### 3.1 Effects of different feeding methods on intestinal microbial diversity

**Figure 1:**
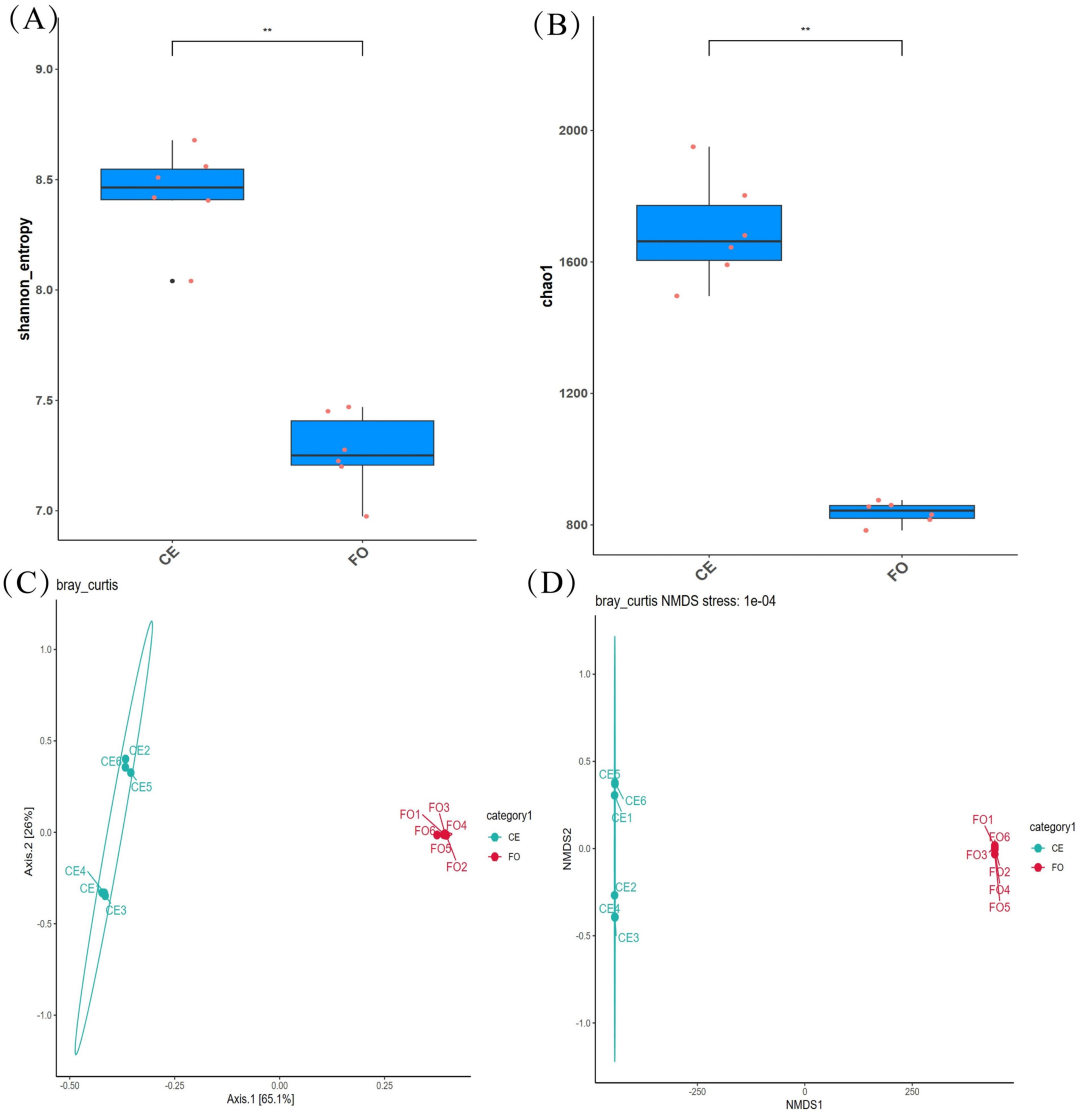
Bacterial diversity analysis. **(A)** Multiple comparisons of the Shannon index between groups. **(B)** Multiple comparisons of the Chao1 index between groups. **(C)** PCoA diagram based on Unweighted UniFrac. **(D)** NMDS analysis diagram.

The data on CE and FO chickens were processed, and the species diversity results were analyzed. As shown in Figure 1, we analyzed the *α*-diversity index, including Shannon (Figure 1A) and Chao1 (Figure 1B). In addition, beta diversity was used to evaluate the differences in microbial community structure between different samples (Figures 1C,D). Alpha diversity analysis revealed significant variations in microbial species diversity and community diversity among different samples. CE chickens exhibited higher levels of microbial richness (Chao1) and diversity (Shannon) compared to FO chickens (*p* < 0.01). Alpha diversity analysis revealed significant variations in microbial species and community diversity among different samples. CE chickens exhibited higher levels of microbial richness and diversity compared to FO chickens (*p* < 0.01). The principal component analysis (PCA) of the samples revealed a distinct separation between chickens under different feeding conditions. Additionally, the non-metric multidimensional scaling (NMDS) plot indicated significant differences in the intestinal microbial community structure of the two types of chickens. Samples under the same conditions showed significant homogeneity. In summary, there are significant differences in intestinal microorganisms between CE chickens and commercial FO chickens. The intestinal microbial community of CE chickens is more diverse, which could be a key factor contributing to the variation in chicken meat quality.

### 3.2 Composition of intestinal microbial communities in CE and FO chickens

**Figure 2:**
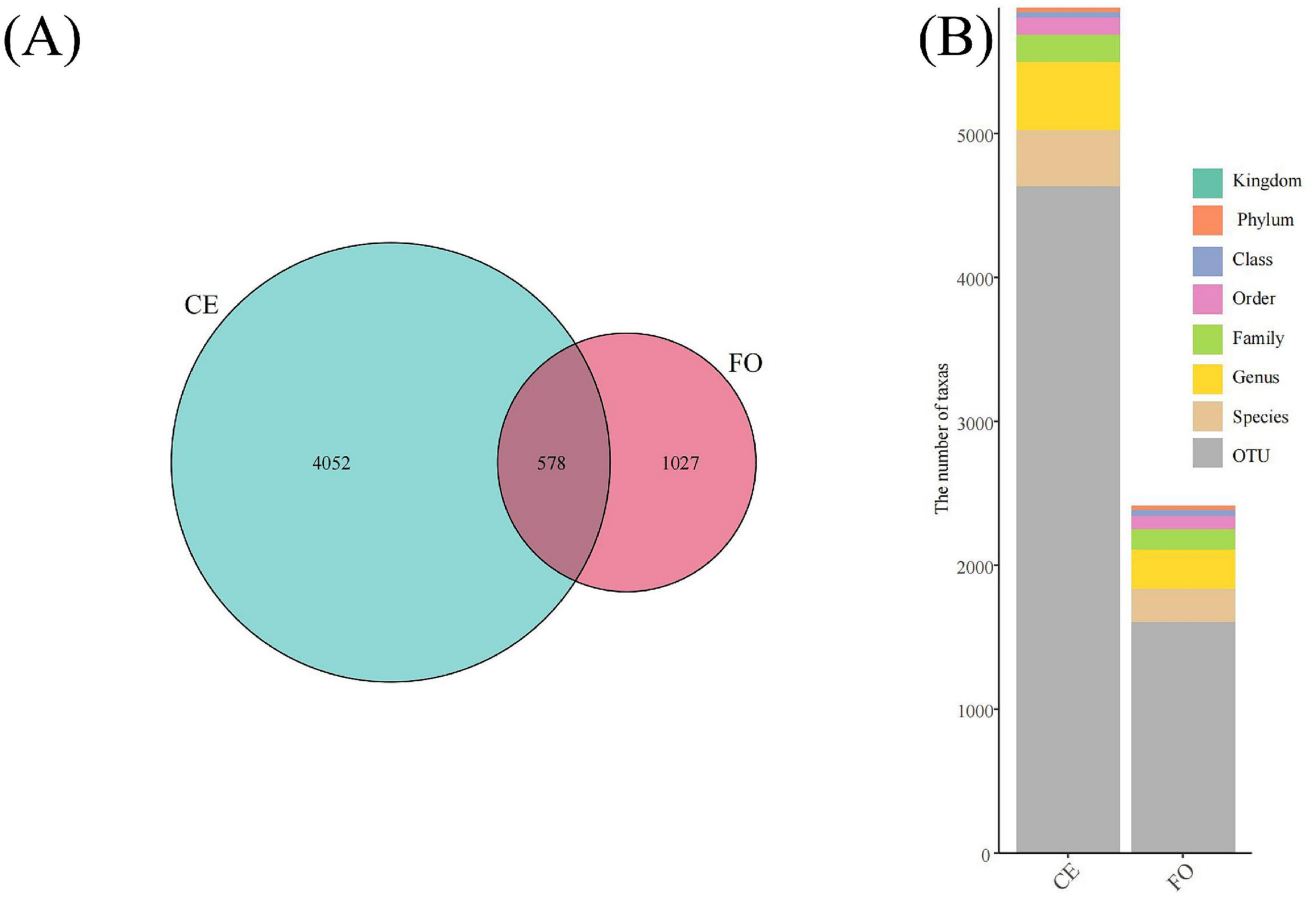
Species diversity analysis based on 16S rRNA sequencing. **(A)** Venn diagram showing common and unique species. **(B)** Annotated bar chart for each sample at every taxonomic level.

**Figure 3:**
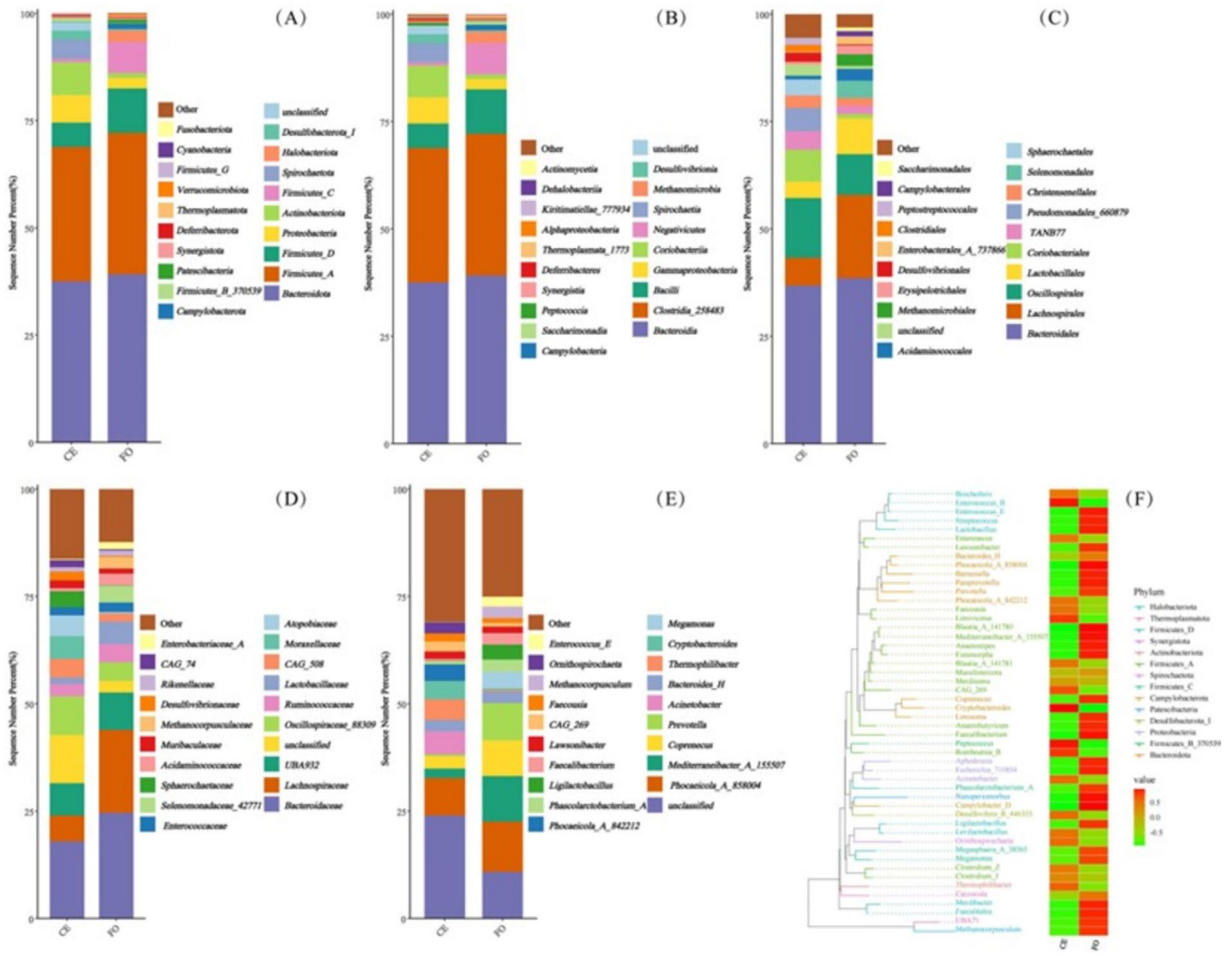
The composition of chicken intestinal microorganisms at different classification levels, including **(A)** phylum, **(B)** class, **(C)** order, **(D)** family, **(E)** genus. **(F)** Phylogenetic evolutionary tree and heatmap showing the distribution of abundance among groups. The left side shows the evolutionary tree, where branches of various colors represent different phyla. Each branch at the end represents an Operational Taxonomic Unit (OTU), and the end is annotated with the genus classification to which the corresponding OTU belongs. If there is no corresponding genus classification, it is represented by “unclassified_genus.” The heat map on the right displays the standardized abundance, where a higher value indicates a higher relative abundance.

The full-length 16S rRNA obtained through synthetic long-read technology was used to analyze the intestinal microbial community of chickens under two different feeding conditions. As shown in Figure 2A, among the 5,657 ASVs obtained after quality filtering, there were 4,052 ASVs unique to CE chickens, 1,027 ASVs unique to FO chickens, and 578 ASVs shared by both types of chickens. At different species levels, the number of taxonomic units (Figure 2B) provides basic information on species annotation statistics. It can be seen that the number of each taxonomic level in CE samples is much higher than in FO samples. Select the representative sequences of the ASVs of interest for phylogenetic analysis. Choose one ASV with the highest abundance as the representative ASV for each genus, and then select the top 50 genera with the highest abundance to draw an evolutionary tree. Combine the absolute abundance of ASVs in each group for heat map visualization. As shown in Figure 3F, the abundance of microbial species in FO chickens is generally higher than that in CE chickens. This reflects the higher species diversity observed in CE chickens, indicating significant differences in the intestinal microorganisms of the two types of chickens.

Representative sequences of ASVs were selected and compared with the Greengenes2 database to obtain species annotation information. Based on the species annotation information, ASVs annotated as chloroplasts, mitochondria, and sequences that could not be classified at the kingdom level were removed. Based on the absolute abundance and annotation information of ASVs, the proportion of sequences in each sample was calculated at seven classification levels: kingdom, phylum, class, order, family, genus, and species. This evaluation method effectively assesses the species annotation resolution of the sample. At the phylum level (Figure 3A), the results showed that the top five chicken intestinal microbial communities were mainly *Bacteroidota*, *Firmicutes_A*, *Firmicutes_D*, *Proteobacteria*, and *Actinobacteriota*. Compared with CE chickens, the relative abundance of *Proteobacteria* and *Actinobacteriota* in FO chickens was lower. At the class level (Figure 3B), the relative abundance of *Gammaproteobacteria* and *Coriobacteriia* in CE chickens was higher than in FO chickens. Among the top five microorganisms (i.e., *Bacteroidia*, *Clostridia_258483*, *Bacilli*, *Coriobacteriia*, and *Gammaproteobacteria*), *Bacteroidia* had the highest relative abundance. At the order level (Figure 3C), *Bacteroidales* had the highest relative abundance. In addition, the composition and abundance of the intestinal microbial community of chickens varied significantly under different feeding conditions. Differences in the intestinal microbial structure and composition between CE and FO chickens were also evident at the family and genus levels (Figures 3D,E). In summary, feeding conditions are key factors that affect the abundance and composition of intestinal microorganisms in chickens.

### 3.3 Differences in intestinal microbiota between CE and FO chickens

**Figure 4:**
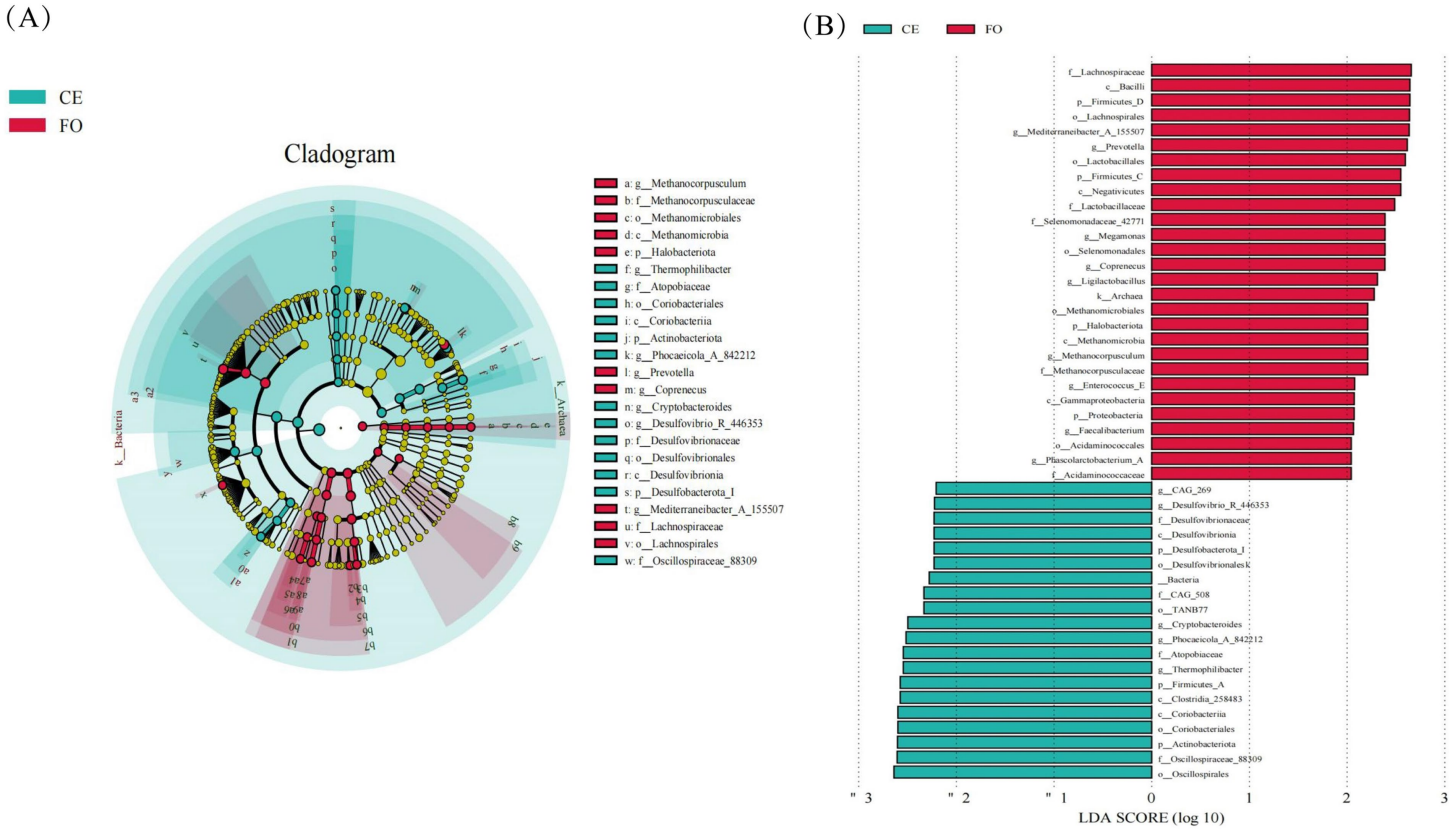
The significance analysis of Operational Taxonomic Unit (OTUs) differences between 16S DNA sequencing groups. **(A)** LEfSe analysis cladogram. From the inside to the outside, the cladogram diagram represents various classification levels such as phylum, order, family, and genus. The lines between the levels represent the affiliation. Each circular node represents a species. If the node is yellow, it indicates that the difference between the groups is not significant. If the node is not yellow, it indicates that the species is a characteristic microorganism of the corresponding color group (with significantly higher abundance in this group). The colored sectors indicate the subordinate taxonomic levels of the characteristic microorganisms. **(B)** LEfSe analysis LDA histogram. Each horizontal column represents a species, and the length of the column corresponds to the LDA value. The higher the LDA value, the greater the difference. The color of the column corresponds to the group to which the species belongs as a characteristic microorganism, and the characteristic microorganisms (biomarkers) indicate a relatively high abundance in the corresponding group.

LEfSe (Linear discriminant analysis Effect Size) analysis is utilized to identify characteristic microorganisms within each group, focusing on those with an LDA (Linear Discriminant Analysis) score exceeding a specified threshold, indicating higher abundance in one group compared to others, as illustrated in Figure 4B. Figure 4A illustrates the taxonomic hierarchy of these characteristic microorganisms simultaneously. In the figure, larger taxa with differences above the family level are indicated with classification intervals and names, while those below the family level are distinguished by color. From this figure, the evolutionary relationships of microorganisms with significant differences between groups can be inferred. Among these microorganisms, 211 distinct Operational Taxonomic Units (OTUs) can be used as key identification indicators. In the CE group, *Oscillospirales*, *Oscillospiraceae_88309*, *Coriobacteriales*, *Coriobacteria*, and *Clostridia_258483*. Conversely, in the FO group, *Lachnospiraceae*, *Bacilli*, *Firmicutes_D*, *Lachnospirales*, *Mediterraneibacter_A_155507*, *Prevotella*, *Lactobacillales*, *Firmicutes*, and *Firmicutes_C* are the most abundant microbial groups. These findings indicate that the gut microbiota of CE chickens is more diverse, suggesting a richer and potentially more complex microbial community. This richness might be attributed to the natural diet and habitat of the CE chickens, which support a diverse microbiome. In contrast, the gut microbiota of FO chickens, shaped by a controlled and nutrient-rich diet, exhibits a distinct microbial composition. Understanding these differences is crucial for comprehending the role of the gut microbiome in health and disease, as well as its potential impact on the overall well-being of the chickens.

### 3.4 Metagenomics analysis of the composition of intestinal microbial communities

**Figure 5:**
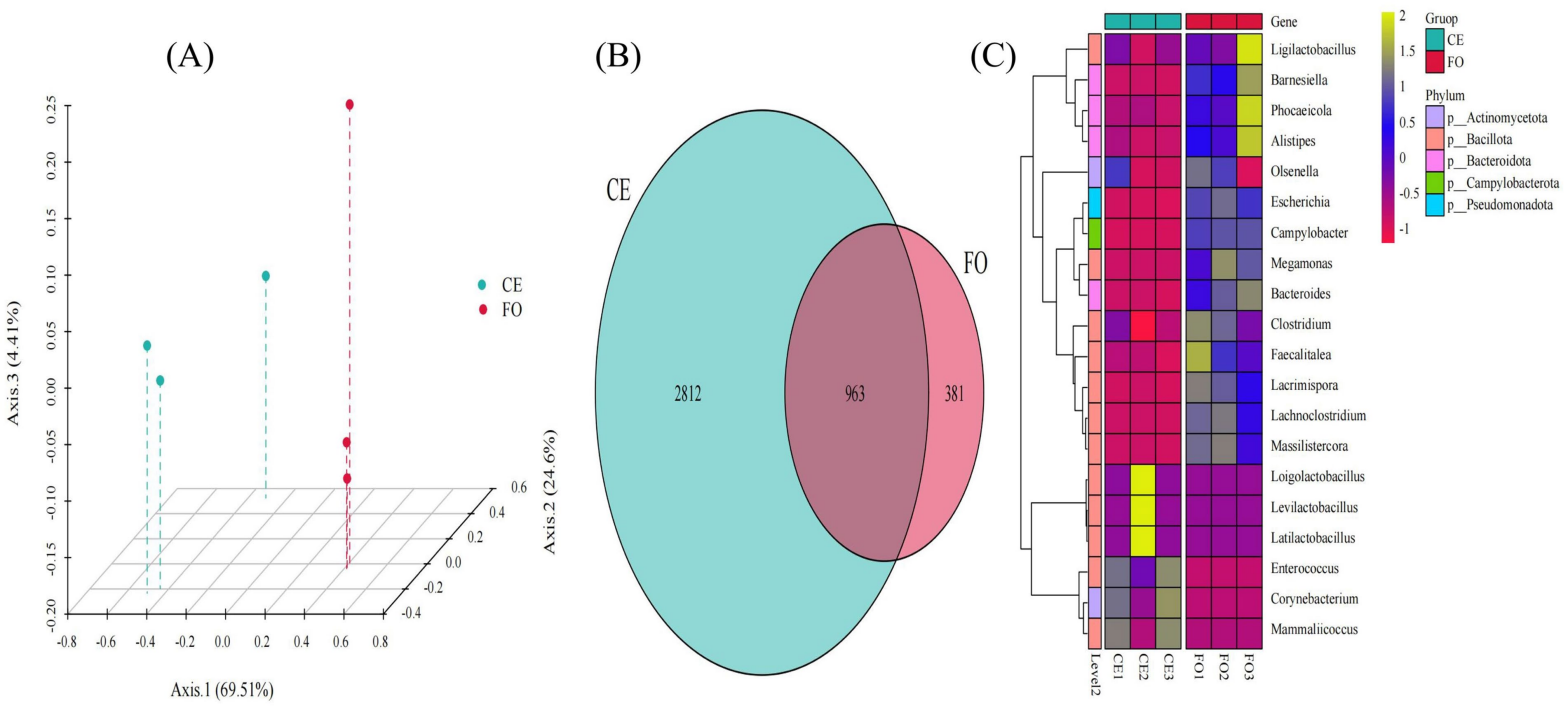
The species composition and diversity of metagenomic analysis. **(A)** PCoA 3D plot based on the Bray-Curtis distance matrix. **(B)** Venn diagram showing common and unique species. **(C)** Genus-level grouping clustering heatmap.

Kraken2 was used to compare a self-built microbial nucleic acid database to calculate the number of sequences of species contained in the sample, while Bracken was used to estimate the actual abundance of species in the sample. Compared with assembly-based species annotation, the reads-based metagenomic species annotation method is more comprehensive and accurate. As shown in Figure 5A, the PCA plot also demonstrates the clustering of CE and FO samples, consistent with the patterns observed in the corresponding 16S amplicon data (Figure 1C). In general, the metagenomic classification is consistent with the 16S amplicon data, and both analyses indicate that variations in feeding conditions lead to significant changes. As shown in Figure 5B, there are 963 species shared by the two groups of samples. There are only 381 unique species in FO, with the number of CE chicken species being significantly greater than that of FO chickens. As expected, in a system with high diversity, the total coverage of these genomes is quite low (~3% of the data read) (Acharya et al., 2023). These species mainly belong to *Actinomycetota*, *Bacillota*, *Bacteroidota, Campylobacterota*, and *Pseudomonadota* (Figure 5C). The abundance of these species in CE chicken samples is significantly higher compared to FO chickens.

### 3.5 Metagenomic analysis reveals metabolic differences in gut microbiota

**Figure 6:**
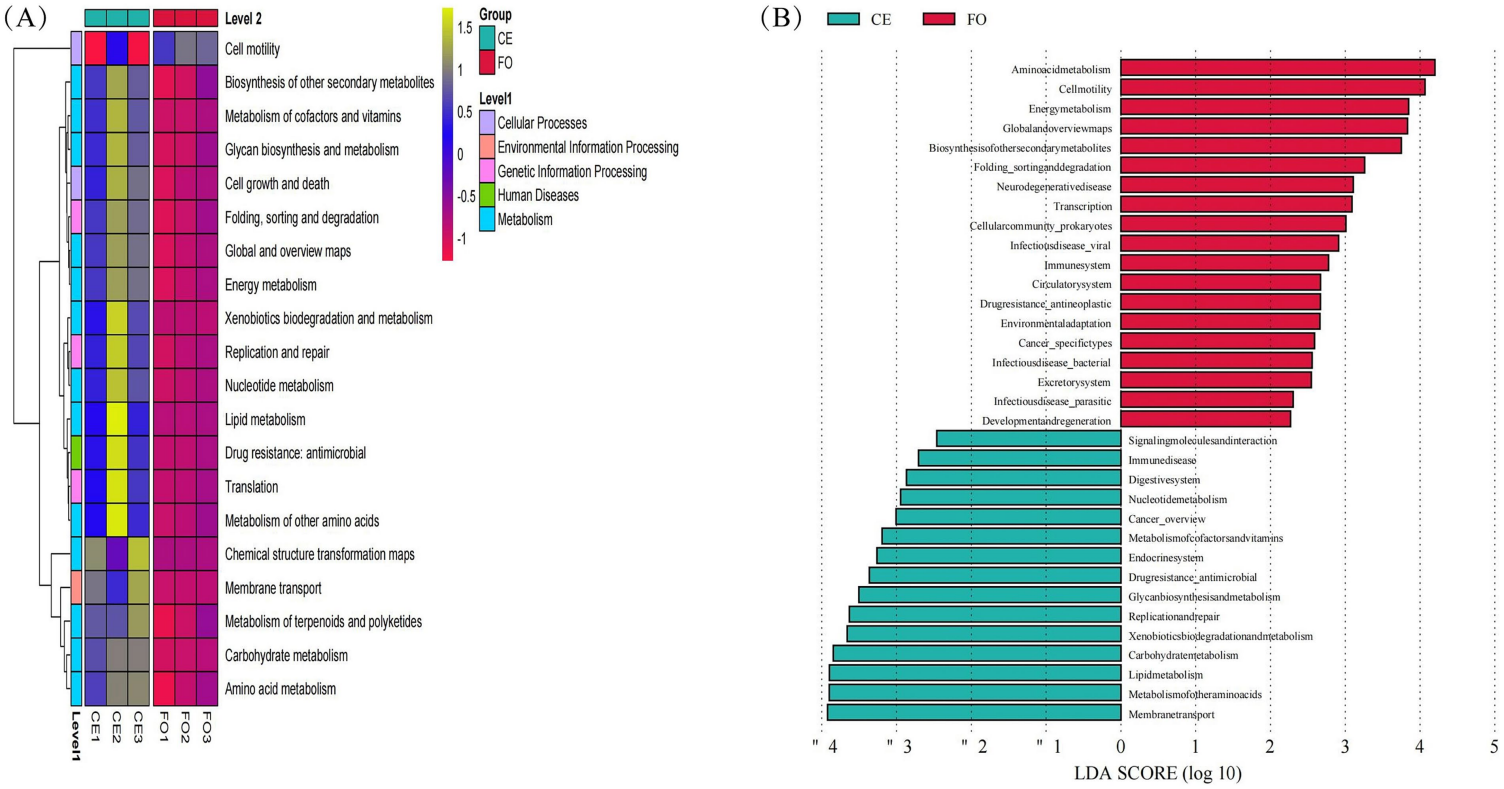
Analysis of functional relative abundance differences. **(A)** Group clustering heat map, **(B)** KEGG basic metabolic pathway LEfSe analysis LDA bar chart.

Six thousand five hundred and forty-five unique KEGG homologous groups (KOs) were annotated in the metagenomes, accounting for 30% of the total predicted proteins in each metagenome. The KO content differed significantly between CE and FO chickens, which were in line with the observations of ASVs (Figure 6B). Among the top 20 differentially abundant KOs identified, 19 were more abundant in CE chicken samples (Figure 6A). These KOs can be broadly categorized into enzymes, transcriptional regulators, or transporters, with a primary focus on metabolic pathways such as membrane transport. Additional pathways encompass amino acid metabolism, lipid metabolism, carbohydrate metabolism, xenobiotics biodegradation, and metabolism. Notably, amino acid metabolism, cell motility, energy metabolism, global and overview maps, and biosynthesis of secondary metabolites were predominant in FO chickens.

**Figure 7:**
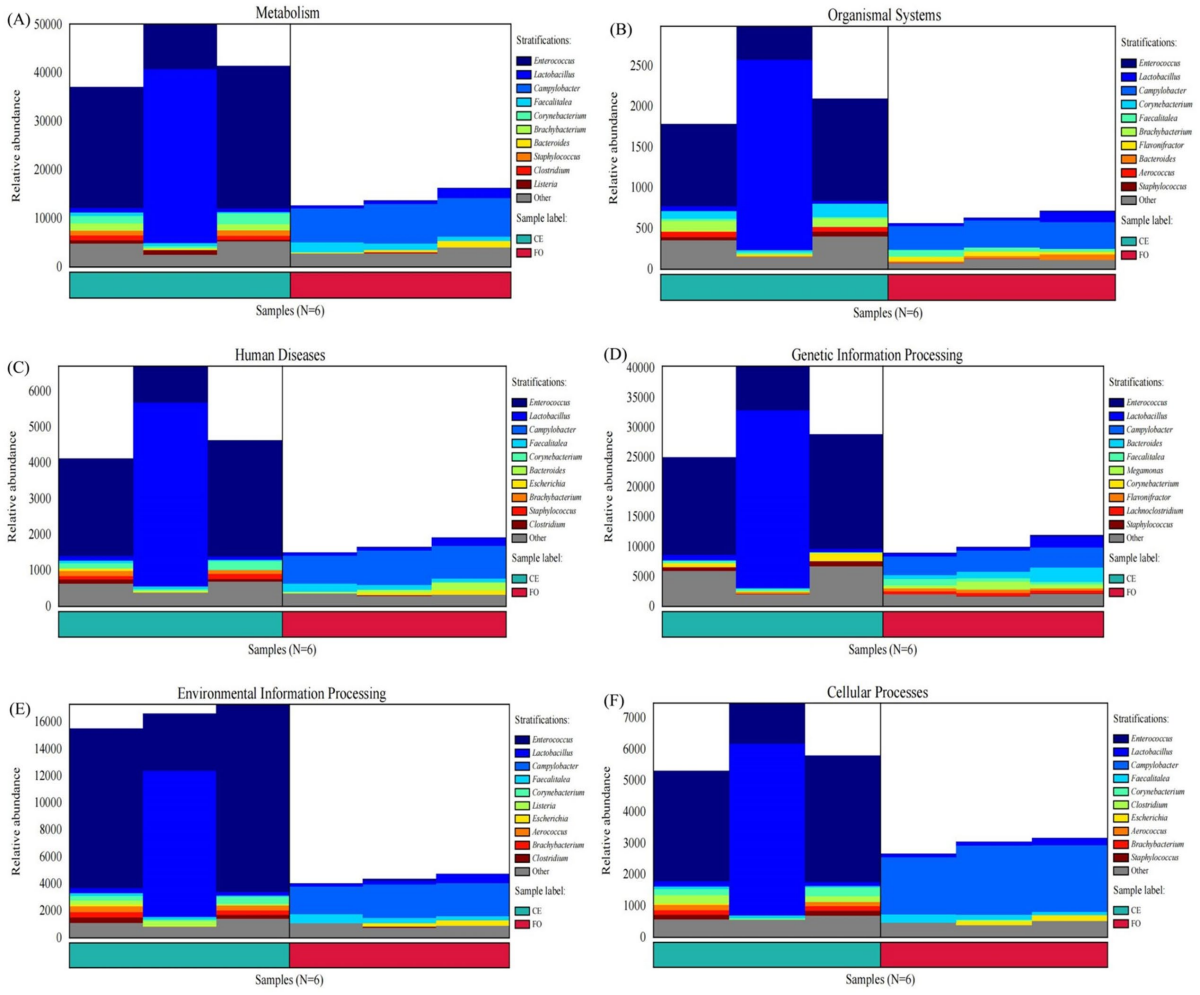
Column chart of species origin of characteristic functions. The horizontal axis corresponds to samples and sample groups, and different groups are marked with different colors. The vertical axis corresponds to the relative abundance of the function in each sample, and different species sources are marked with different colors. **(A)** Metabolism. **(B)** Organismal Systems. **(C)** Human Diseases. **(D)** Genetic Information Processing. **(E)** Environmental Information Processing. **(F)** Cellular Processes.

We identified the species sources of the functions based on the six characteristic functions. As shown in Figure 7, the primary species contributing to the differences in intestinal microbial metabolism between CE and FO chickens were identified as *Enterococcus*, *Lactobacillus*, *Campylobacter*, *Faecalitalea*, and *Corynebacterium*. Among these, *Enterococcus* emerged as the most significant influencing factor. The abundance of *Enterococcus* in CE samples was significantly higher than in FO samples, emphasizing its crucial role in distinguishing the microbial metabolic profiles of the two groups. Other strains, such as disease-associated *Campylobacter* content, were significantly higher in the FO group than in the CE group. *Lactobacillus, Faecalitalea*, and *Cornebacterium* further fuel the unique microbial ecology between the two groups. In addition, changes in major microorganisms also led to differences in Metabolism, Organismal Systems, Human Diseases, Genetic Information Processing, Environmental Information Processing and Cellular Processes between CE and FO groups. These findings highlight the complex relationship between diet, microbial composition, and metabolic function in the gut ecosystem.

## 4 Discussion

This study aimed to explore the composition and functional differences in the intestinal microbiota of CE and FO through 16 s rRNA gene sequencing and metagenomic analysis, and the impact of these differences on chicken quality. The research results show that there are significant differences in the intestinal microbial diversity and abundance of specific bacterial groups between CE and FO. In particular, the intestinal microbial diversity of CE is significantly higher than that of FO, and the abundance of some key microbial groups such as Enterococcus is significantly higher in CE. The intestinal microbiota of CE group was more abundant, possibly because CE group was fed mainly on rice, which contains dietary fiber, starch and non-starch polysaccharides, which promote the growth of beneficial bacteria, increase microbial diversity, and provide additional nutrients (Makki et al., 2018). However, the fiber and nutrients in the feed fed by FO group are relatively single, which leads to poor abundance and diversity of intestinal microorganisms in FO group. The intestinal microbial community of CE group was more diverse, which may affect chicken quality. Dietary fiber and non-starch polysaccharide in the rice fed by the main CE group provided rich nutrition for microorganisms and promoted their diversity (Jha and Mishra, 2021). The fermentation of starch produces short-chain fatty acids, which are beneficial to microorganisms (Mann et al., 2024). In addition, the vitamins and minerals in rice may also have a positive impact on the microbiome (Dewan et al., 2023). Together, these factors may lead to changes in chicken quality by altering microbial metabolites, flavor and texture. The content of this part has been supplemented in the discussion section of the article. These research results provide important insights for a deeper understanding of the impact of intestinal microorganisms in CE and FO on chicken quality, and provide possible ideas and methods for optimizing chicken production.

Through LEfSe analysis, we identified several significantly different characteristic microbial groups. In the CE group, the abundance of microorganisms such as *Oscillospirales*, *Oscillospiraceae_88309*, *Coriobacteriales*, and *Actinobacteriota* was significantly higher; while in the FO group, the abundance of microorganisms such as *Lachnospiraceae*, *Bacilli*, *Firmicutes_D*, *Lachnospirales*, *Mediterraneanibacter_A_155507*, *Prevotella*, *Lactobacilales*, *Firmicutes*, and *Firmicutes_C*, etc. Microorganisms are richer. Metagenomic functional analysis showed that the metabolic functions of intestinal microorganisms were significantly different between the two groups of chickens. The high abundance of *Enterococcus* in CE may be associated with enhanced immune function and better intestinal health, which may directly affect the meat quality and overall health of organic chickens (Zhang et al., 2024; Li et al., 2024). Studies have shown that the presence of avian *Enterococci* is closely related to the growth performance and meat quality characteristics of birds (Aruwa et al., 2021; Souillard et al., 2022; Shang et al., 2018). *Enterococci* play an important role in the microbiome, especially when it comes to gut health. On the one hand, an appropriate abundance of avian enterococci helps promote the growth and development of birds and may have a positive impact on the mouthfeel, texture, and flavor of the meat. On the other hand, *Enterococci* are also able to survive in complex environments due to their drug resistance and are essential for maintaining ecological balance (Shao et al., 2024). Different sample sources, environmental conditions, and handling and storage methods can affect the abundance of *Enterococcus*, so understanding its specific role and influencing factors is important for evaluating sample quality and handling methods. Therefore, the meat quality of the CE group is more popular among people, which may be related to the abundance of *enterobacteriaceae*. In addition, the presence of beneficial bacteria such as *Lactobacillus* may contribute to better digestion and nutrient absorption, thereby affecting the taste and nutritional value of the meat (Dong et al., 2024; Gao et al., 2022; Abd El-Hack et al., 2020). Firstly, *Lactobacillus* can produce various digestive enzymes, such as proteases, lipases, and amylases. These enzyme substances can directly act on the large molecular nutrients in food, breaking down proteins into amino acids, fats into fatty acids and glycerol, starch into small molecule substances such as glucose, making these nutrients that were originally difficult to directly absorb and utilize in the chicken intestinal tract absorbable, greatly improving the digestibility of nutrients in feed. Secondly, *Lactobacillus* can regulate the intestinal microbiota environment. They compete with harmful bacteria for adhesion sites and nutrients in intestinal epithelial cells, inhibit the growth and reproduction of harmful bacteria, reduce the damage of harmful bacteria and their toxins to intestinal mucosa, and maintain the integrity of intestinal mucosa. A complete and healthy intestinal mucosa is crucial for the absorption of nutrients, as it ensures the smooth passage of nutrients through intestinal epithelial cells into the bloodstream.

The results indicated that different diets impacted the intestinal flora structure of chickens. Variations between the CE and FO diets could influence the production of intestinal microbial metabolites, subsequently affecting the taste, texture, and nutritional value of the chicken (Jha and Mishra, 2021; Li et al., 2024; Shen et al., 2024). The intestinal flora plays a crucial role in poultry performance, influencing nutrient absorption, immune system function, metabolite production, and stress response (Aruwa et al., 2021). A healthy gut microbiota enhances nutrient utilization efficiency, strengthens immune defenses, produces beneficial metabolites, and mitigates stress responses, thereby improving growth and weight. Effective management of the gut microbiota provides a scientific foundation for chicken production, including optimizing feed formulations through microbiota monitoring, controlling environmental hygiene, reducing stressors, and implementing personalized management strategies to enhance chicken health and productivity (Cai et al., 2024). By in-depth research on these influencing factors, a scientific basis can be provided for the development of a healthier and higher-quality poultry farming industry.

This study revealed significant differences in intestinal microbiota and functions between CE and FO, but there are still some limitations in understanding the relationship between intestinal microorganisms and chicken quality. First, the sample size of the existing studies is small, and future studies need to increase the number of samples and extend the follow-up period to verify the stability and persistence of these differences in different groups. Secondly, relying solely on 16 s rRNA gene and metagenomic analysis may not fully reveal the complex relationship between intestinal microorganisms and chicken quality. Future research can combine multiple analysis methods such as metabolomics and proteomics to more comprehensively and deeply analyze the specific impact mechanism of intestinal microorganisms on chicken quality. In addition, future studies should consider the impact of factors such as different regions and seasons on the intestinal flora of chickens (Shi et al., 2019; Sekelja et al., 2012; Wang et al., 2016). Environmental conditions and feeding patterns in different regions may have differential effects on the intestinal microbiota of poultry, so consideration of these factors can further improve the understanding of factors affecting intestinal microbiota. By comprehensively considering region, season, and other environmental factors, the impact of intestinal microorganisms on chicken quality can be more comprehensively and accurately assessed, providing more scientific guidance for poultry feeding management and product quality. The development of these in-depth studies will help to better understand and exploit the mechanisms of the impact of poultry intestinal microbiota on chicken quality.

## 5 Conclusion

The results of 16 s rRNA gene sequencing analysis showed that there were significant differences in intestinal microbial diversity and specific microflora abundance between CE and FO chickens. The intestinal microbial diversity of CE chickens was significantly higher than that of FO chickens, and the abundance of key microflora such as *Enterococcus* was significantly higher in CE chickens. The analysis of metagenomic function showed that there were significant differences in intestinal microbial metabolic function between the two groups. The existence of beneficial bacteria such as *Enterococcus* and *Lactobacillus* may directly affect the meat quality and overall health of CE chickens. As a result of obtaining different diets, the diversity and functional of intestinal microbiota of CE chickens affects the taste, texture, and nutritional value of chicken meat. Although 16 s rRNA gene and macrogenomic analysis provide us with important insights, a single method still has its limitations. Future research needs to integrate more technical tools, such as metabonomics and proteomics, to obtain more comprehensive conclusions.

## Data Availability

The data presented in the study are deposited in the NCBI repository. Available at: https://www.ncbi.nlm.nih.gov/sra/PRJNA1171564, accession number PRJNA1171564.

## Footnotes

^1^ http://www.bioincloud.tech

## References

[ref1] Abd El-Hack M. E. El-Saadony M. T. Shafi M. E. Qattan S. Y. A. Batiha G. E. Khafaga A. F.. (2020). Probiotics in poultry feed: a comprehensive review. J. Anim. Physiol. Anim. Nutr. 104, 1835–1850. doi: 10.1111/jpn.13454, PMID: 32996177

[ref2] Acharya S. M. Yee M. O. Diamond S. Andeer P. F. Baig N. F. Aladesanmi O. T.. (2023). Fine scale sampling reveals early differentiation of rhizosphere microbiome from bulk soil in young Brachypodium plant roots. ISME Commun. 3:54. doi: 10.1038/s43705-023-00265-1, PMID: 37280433 PMC10244434

[ref3] Al-Khalaifah H. Al-Nasser A. (2021). Dietary source of polyunsaturated fatty acids influences cell cytotoxicity in broiler chickens. Sci. Rep. 11:10113. doi: 10.1038/s41598-021-89381-3, PMID: 34001928 PMC8129153

[ref4] Aruwa C. E. Pillay C. Nyaga M. M. Sabiu S. (2021). Poultry gut health – microbiome functions, environmental impacts, microbiome engineering and advancements in characterization technologies. J. Anim. Sci. Biotechnol. 12:119. doi: 10.1186/s40104-021-00640-9, PMID: 34857055 PMC8638651

[ref5] Bernard M. Lecoeur A. Coville J.-L. Bruneau N. Jardet D. Lagarrigue S.. (2024). Relationship between feed efficiency and gut microbiota in laying chickens under contrasting feeding conditions. Sci. Rep. 14:8210. doi: 10.1038/s41598-024-58374-3, PMID: 38589474 PMC11001975

[ref6] Borda-Molina D. Seifert J. Camarinha-Silva A. (2018). Current perspectives of the chicken gastrointestinal tract and its microbiome. Comput. Struct. Biotechnol. J. 16, 131–139. doi: 10.1016/j.csbj.2018.03.002, PMID: 30026889 PMC6047366

[ref7] Cai K. Liu R. Wei L. Wang X. Cui H. Luo N.. (2024). Genome-wide association analysis identify candidate genes for feed efficiency and growth traits in Wenchang chickens. BMC Genomics 25:645. doi: 10.1186/s12864-024-10559-w, PMID: 38943081 PMC11212279

[ref8] Chen L. Bai X. Wang T. Liu J. Miao X. Zeng B.. (2023). Gut microbial diversity analysis of different native chickens and screening of chicken-derived probiotics. Animals 13:3672. doi: 10.3390/ani13233672, PMID: 38067023 PMC10705773

[ref9] Coletta L. D. Pereira A. L. Coelho A. A. D. Savino V. J. M. Menten J. F. M. Correr E.. (2012). Barn vs. free-range chickens: differences in their diets determined by stable isotopes. Food Chem. 131, 155–160. doi: 10.1016/j.foodchem.2011.08.051

[ref10] Dai H. Han J. Wang T. Yin W.-B. Chen Y. Liu H. (2023). Recent advances in gut microbiota-associated natural products: structures, bioactivities, and mechanisms. Nat. Prod. Rep. 40, 1078–1093. doi: 10.1039/D2NP00075J, PMID: 37013809

[ref11] Dewan M. F. Ahiduzzaman M. Islam M. N. Shozib H. B. (2023). Potential benefits of bioactive compounds of traditional Rice grown in south and Southeast Asia: a review. Rice Sci. 30, 537–551. doi: 10.1016/j.rsci.2023.07.002

[ref12] Ding S. Chang J. Zhang W. Ji S. Chi Y. (2024). Environmental microbial diversity and water pollution characteristics resulted from 150 km coastline in Quanzhou Bay offshore area. Front. Microbiol. 15:1438133. doi: 10.3389/fmicb.2024.1438133 39027103 PMC11254811

[ref13] Dong S. Li L. Hao F. Fang Z. Zhong R. Wu J.. (2024). Improving quality of poultry and its meat products with probiotics, prebiotics, and phytoextracts. Poult. Sci. 103:103287. doi: 10.1016/j.psj.2023.103287, PMID: 38104412 PMC10966786

[ref14] Feng Y. Zhang M. Liu Y. Yang X. Wei F. Jin X.. (2023). Quantitative microbiome profiling reveals the developmental trajectory of the chicken gut microbiota and its connection to host metabolism. iMeta 2:e105. doi: 10.1002/imt2.105, PMID: 38868437 PMC10989779

[ref15] Franzosa E. A. McIver L. J. Rahnavard G. Thompson L. R. Schirmer M. Weingart G.. (2018). Species-level functional profiling of metagenomes and metatranscriptomes. Nat. Methods 15, 962–968. doi: 10.1038/s41592-018-0176-y, PMID: 30377376 PMC6235447

[ref16] Gao H. Li X. Chen X. Hai D. Wei C. Zhang L.. (2022). The functional roles of *Lactobacillus acidophilus* in different physiological and pathological processes. J. Microbiol. Biotechnol. 32, 1226–1233. doi: 10.4014/jmb.2205.05041, PMID: 36196014 PMC9668099

[ref17] Guan Z. Lin D. Chen D. Guo Y. Lu Y. Han Q.. (2022). Soil microbial communities response to different fertilization regimes in young *Catalpa bungei* plantation. Front. Microbiol. 13:948875. doi: 10.3389/fmicb.2022.948875 36118227 PMC9473346

[ref18] Jha R. Mishra P. (2021). Dietary fiber in poultry nutrition and their effects on nutrient utilization, performance, gut health, and on the environment: a review. J. Anim. Sci. Biotechnol. 12:51. doi: 10.1186/s40104-021-00576-0, PMID: 33866972 PMC8054369

[ref19] Kers J. G. Velkers F. C. Fischer E. A. J. Hermes G. D. A. Stegeman J. A. Smidt H. (2018). Host and environmental factors affecting the intestinal microbiota in chickens. Front. Microbiol. 9:235. doi: 10.3389/fmicb.2018.00235 29503637 PMC5820305

[ref20] Li J. Li Y. Xiao H. Li W. Ye F. Wang L.. (2024). The intestinal microflora diversity of aboriginal chickens in Jiangxi province, China. Poult. Sci. 103:103198. doi: 10.1016/j.psj.2023.103198, PMID: 38016408 PMC10696398

[ref21] Makki K. Deehan E. C. Walter J. Bäckhed F. (2018). The impact of dietary Fiber on gut microbiota in host health and disease. Cell Host Microbe 23, 705–715. doi: 10.1016/j.chom.2018.05.012, PMID: 29902436

[ref22] Mann E. R. Lam Y. K. Uhlig H. H. (2024). Short-chain fatty acids: linking diet, the microbiome and immunity. Nat. Rev. Immunol. 24, 577–595. doi: 10.1038/s41577-024-01014-8 38565643

[ref23] Minas K. McEwan N. R. Newbold C. J. Scott K. P. (2011). Optimization of a high-throughput CTAB-based protocol for the extraction of qPCR-grade DNA from rumen fluid, plant and bacterial pure cultures. FEMS Microbiol. Lett. 325, 162–169. doi: 10.1111/j.1574-6968.2011.02424.x, PMID: 22029887

[ref24] Oviedo-Rondón E. O. (2019). Holistic view of intestinal health in poultry. Anim. Feed Sci. Technol. 250, 1–8. doi: 10.1016/j.anifeedsci.2019.01.009

[ref25] Sekelja M. Rud I. Knutsen S. H. Denstadli V. Westereng B. Næs T.. (2012). Abrupt temporal fluctuations in the chicken fecal microbiota are explained by its gastrointestinal origin. Appl. Environ. Microbiol. 78, 2941–2948. doi: 10.1128/AEM.05391-11, PMID: 22307311 PMC3318845

[ref26] Shang Y. Kumar S. Oakley B. Kim W. K. (2018). Chicken gut microbiota: Importance and detection technology. Front Vet Sci 5:254. doi: 10.3389/fvets.2018.00254 30406117 PMC6206279

[ref27] Shao Y. Wang S. Xu X. Sun C. Cai F. Guo Q.. (2024). Non-specific elevated serum free fatty acids in lung Cancer patients: nutritional or pathological? Nutrients 16:2884. doi: 10.3390/nu16172884, PMID: 39275200 PMC11396813

[ref28] Shen H. Wang T. Dong W. Sun G. Liu J. Peng N.. (2024). Metagenome-assembled genome reveals species and functional composition of Jianghan chicken gut microbiota and isolation of Pediococcus acidilactic with probiotic properties. Microbiome 12:25. doi: 10.1186/s40168-023-01745-1, PMID: 38347598 PMC10860329

[ref29] Shi S. Qi Z. Gu B. Cheng B. Tu J. Song X.. (2019). Analysis of high-throughput sequencing for cecal microbiota diversity and function in hens under different rearing systems. 3 Biotech 9:438. doi: 10.1007/s13205-019-1970-7 PMC6834809 31750036

[ref30] Souillard R. Laurentie J. Kempf I. Le Caër V. Le Bouquin S. Serror P.. (2022). Increasing incidence of Enterococcus-associated diseases in poultry in France over the past 15 years. Vet. Microbiol. 269:109426. doi: 10.1016/j.vetmic.2022.109426, PMID: 35526479

[ref31] Stanley D. Hughes R. J. Moore R. J. (2014). Microbiota of the chicken gastrointestinal tract: influence on health, productivity and disease. Appl. Microbiol. Biotechnol. 98, 4301–4310. doi: 10.1007/s00253-014-5646-2 24643736

[ref32] Sun H. Liu P. Nolan L. K. Lamont S. J. (2016). Thymus transcriptome reveals novel pathways in response to avian pathogenic *Escherichia coli* infection. Poult. Sci. 95, 2803–2814. doi: 10.3382/ps/pew202, PMID: 27466434 PMC5144662

[ref33] Sun J. Wang Y. Li N. Zhong H. Xu H. Zhu Q.. (2018). Comparative analysis of the gut microbial composition and meat flavor of two chicken breeds in different rearing patterns. Biomed. Res. Int. 2018, 1–13. doi: 10.1155/2018/4343196 PMC6206517 30410932

[ref34] Wang L. Lilburn M. Yu Z. (2016). Intestinal microbiota of broiler chickens as affected by litter management regimens. Front. Microbiol. 7:593. doi: 10.3389/fmicb.2016.00593 27242676 PMC4870231

[ref35] Young J. C. Zhou T. Yu H. Zhu H. Gong J. (2007). Degradation of trichothecene mycotoxins by chicken intestinal microbes. Food Chem. Toxicol. 45, 136–143. doi: 10.1016/j.fct.2006.07.028, PMID: 17011105

[ref36] Zhang Y. Liu Y. Jiao S. Wang Y. Sa R. Zhao F.. (2024). Short-term supplementation with uncoated and encapsulated *Enterococcus faecium* affected growth performance, gut microbiome and intestinal barrier integrity in broiler chickens. Poult. Sci. 103:103808. doi: 10.1016/j.psj.2024.103808, PMID: 38761463 PMC11133978

[ref37] Zhou X. Jiang X. Yang C. Ma B. Lei C. Xu C.. (2016). Cecal microbiota of Tibetan chickens from five geographic regions were determined by 16S rRNA sequencing. MicrobiologyOpen 5, 753–762. doi: 10.1002/mbo3.367, PMID: 27139888 PMC5061713

